# Placental telomere length shortening is not associated with severe preeclampsia but the gestational age

**DOI:** 10.18632/aging.204445

**Published:** 2022-12-27

**Authors:** Xiaotong Yang, Paula A. Benny, Elorri Cervera-Marzal, Biyu Wu, Cameron B. Lassiter, Joshua Astern, Lana X. Garmire

**Affiliations:** 1 Department of Computational Medicine and Bioinformatics, University of Michigan, Ann Arbor, MI 48109, USA; 2 Epidemiology Program, University of Hawaii Cancer Center, Honolulu, HI 96813, USA; 3 Department of Nutritional Science, University of Hawaii, Honolulu, HI 96822, USA; 4 University of Hawai’i Biorepository John A. Burns School of Medicine, University of Hawaii at Manoa, Honolulu, HI 96813, USA

**Keywords:** pregnancy, placenta aging, telomere length, preeclampsia, placenta

## Abstract

Variations in telomere length (TL) have been associated with aging, stress, and many diseases. Placenta TL is an essential molecular component influencing the outcome of birth. Previous investigations into the relationship between placenta TL and preeclampsia (PE) have produced conflicting findings. We conducted a retrospective case-control analysis in this study to address the disparity. We used placenta samples from 224 births received from Hawaii Biorepository (HiBR) between 2006 and 2013, comprising 129 healthy full-term controls and 95 severe PE samples. The average absolute placental TL was calculated using the quantitative polymerase chain reaction (qPCR) technique. We utilized multiple linear regressions to associate placental TL with severe PE and other demographic, clinical and physiological data. The outcome demonstrates that the placental TL of severe PE cases did not significantly differ from that of healthy controls. Instead, there is a strong correlation between gestational age and placenta TL shortening. Placental TL also exhibits racial differences: (1) Latino moms’ TL is significantly longer than non-Latino mothers’ (p=0.009). (2) Caucasian patients with severe PE have shorter TL than non-Caucasian patients (p=0.0037). This work puts the long-standing question of whether severe PE influences placental TL to rest. Placental TL is not related to severe PE but is negatively associated with gestational age and is also affected by race.

## INTRODUCTION

Preeclampsia (PE) is a pregnancy syndrome that affects women after 20 weeks of pregnancy, characterized by new-onset gestational hypertension and proteinuria [1]. Other clinical symptoms include renal failure, cardiac malfunction or arrest, stroke, and possibly maternal and fetal death [1]. PE affects between 2-8 percent of pregnant women worldwide, and 3.1 percent in the United States [2, 3]. It is one of the most common causes of pregnancy-related death and is linked to a variety of long-term mother and fetal morbidities. PE in prior pregnancies, chronic hypertension, multiple gestations, higher body mass index (BMI), pregestational diabetes, and antiphospholipid syndrome are all major risk factors for PE [4]. PE is a complex and heterogeneous disorder with multiple subtypes: PE can be classified as mild, severe, or superimposed based on the severity of the symptoms [1]; PE that appears before week 34 of pregnancy is called early-onset PE, and PE that appears after week 34 is called late-onset PE [5]. Although epigenetic, transcriptomic, proteomic, and metabolic methods have increasingly been used in PE research, the molecular mechanisms behind these symptoms remain unknown [6, 7].

Telomeres consist of repeated DNA sequences and protective protein complexes. They function as the protective structures at chromosome ends [8]. Telomeres shorten continuously in each cell division and the process may be accelerated by chronic diseases and environmental stress [9]. TL has been long considered a biomarker for aging [10]. In addition, abnormal TL has been linked with an increased risk of developing various diseases, such as diabetes, cardiovascular disease and chronic kidney disease [11–13]. Placentas play a central role in PE, and it is believed that the onset of PE originated from defective placentation and the release of antiangiogenic factors from the placenta into the maternal circulation [4]. Previous epigenetic studies have suggested accelerated aging in placentas from early-onset PE [14]. Therefore, it is of great interest to investigate if such presumably “pre-aging” stress exerted in placenta epigenetics is also manifested in the form of TL shortening. However, in this regard controversy abounds in multiple earlier studies [15–18]. We speculate that other factors may significantly confound placenta TL interpretation in these studies, such as maternal age, gestational age, ethnicity, and pregnancy history.

To address if these factors might have contributed to the apparently conflicting conclusions, we here conducted the largest multiethnic cohort (MEC) study so far, containing placentas of 95 severe PE and 129 matched full-term deliveries in Honolulu, Hawaii between January 2006 and June 2013. Previously, with a subset of matched maternal blood samples of this cohort, we had reported potential lipidomic biomarkers for severe PE [19]. Here we measured the total placental TL by qPCR technology and then conducted linear regression analysis between placental TL and physiological and clinical factors. Our objectives are: (1) studying if there is indeed an association between severe PE and placental TL shortening; (2) revealing other potential maternal or fetal factors associated with placental TL. This study would provide evidence, or the lack of it, for the link between PE and placental aging through altered TL length.

## RESULTS

### Overview of the study cohort

**Figure 1:**
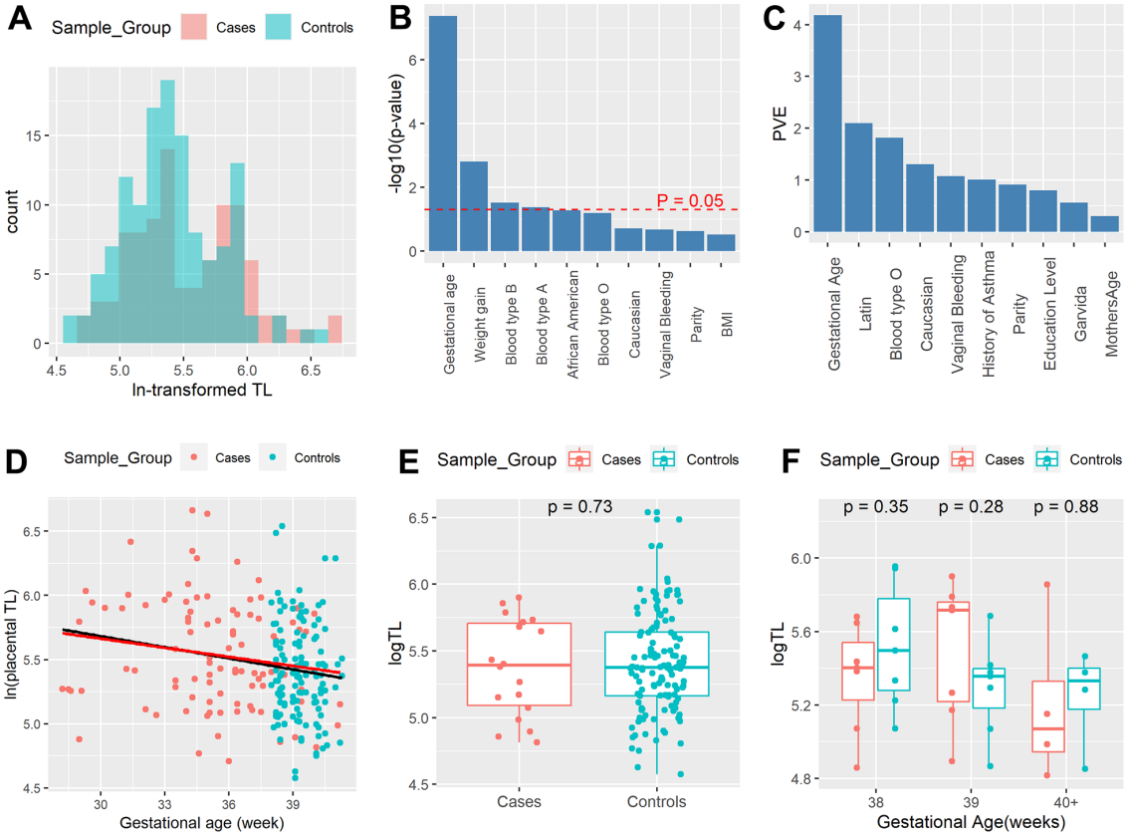
**The lack of association between severe preeclampsia (PE) and telomere length (TL) after stratifying by gestational age.** (**A**) Distribution of ln-transformed relative placental TL in the third trimester. Red color: severe PE samples; Blue color: control samples. (**B**) Severe PE is highly confounded by clinical variables. Top 10 most relevant phenotypic variables of severe PE, calculated using logistic regression. (**C**) Top 10 variables with the highest proportion of variance explained (PVE), per ANOVA analysis. (**D**) Scatter plot of TL by delivery gestational age, with red dots representing severe PE cases and blue dots representing controls. The black line represents linear regression of TL on gestational age using all samples; the red line shows the linear regression of TL on gestational age using only severe PE cases. (**E**) Boxplots of placenta TL of term severe PE samples (n=18) and term controls (n=133). (**F**) Boxplots of placenta TL of term severe PE samples (n=18) and the subset of matched term controls (n=18) stratified by gestational age (38, 39, 40+ weeks).

In Table 1, we present the phenotypic characteristics of 95 pregnancies with severe PE and 129 healthy controls that are matched in criteria including maternal age, height, BMI, blood type, ethnicity and weight gain between the severe PE cases and controls. There is no significant difference in the above-mentioned factors between the severe PE cases and controls, confirming the success of matching criteria in the study design. We excluded samples from smokers and samples with neonatal malformations. All patients with gestational diabetes mellitus (GDM) were found in the case group, so we also removed GDM samples from the analysis. From the correlation heatmap of all phenotypic variables (Supplementary Figure 1), significantly more mothers from the severe PE group have other pre-existing conditions including history of anemia (p = 0.0046) and chronic hypertension (p = 0.0013) or comorbidities like intrauterine growth restriction (IUGR) (p=4.04e-7) and ruptured membrane (p=0.0089). More control samples have macrosomia (p=0.031) than severe PE cases. As expected, the severe PE group has a smaller delivery gestational age (p < 4.05e-23): the average and standard deviation of the delivery gestational age in severe PE cases is 35.28 weeks +/- 3.01 weeks, compared to controls 39.33 weeks +/- 0.85. The distribution of absolute TL is right-skewed (Supplementary Figure 2), similar to observations of TL in other tissues [20]. We ln-transformed TL similar to others [21, 22], so that the TL is approximately Gaussian distribution and suitable for the downstream linear regression analysis. All the TL in the remaining report refers to ln-transformed TL unless noted otherwise. The average Ln-transformed TL in severe PE cases is 5.53, significantly higher than the average value of TL in controls 5.41 (p = 0.018) (Figure 1A).

**Table 1:** Differences in demographic and clinical variables between severe PE cases and controls.

**Variables**	**Preeclampsia (n = 95)**	**Control (n = 129)**	p-value*
	**Mean(sd)**	**Mean(sd)**	**(Two group)**
**Gestational Age (week)**	35.28(3.01)	39.33 (0.85)	4.05e-23 ***
**Mother’s Age**	29.87(6.64)	29.53(6.53)	NS
**Weight Gain (lbs)**	35.79(20.29)	31.80(16.21)	NS
**Mother’s Height**	5.23(0.45)	5.30(0.38)	NS
**Pre-pregnancy BMI**	28.53(7.66)	28.01(6.18)	NS
**Blood Type (n)**			NS
A	27.00	43.00	-
AB	10.00	9.00	-
B	23.00	26.00	-
O	35.00	51.00	-
**Ethnicity (n)**			NS
African American	2.00	2.00	-
Asian	45.00	72.00	-
Caucasian	14.00	17.00	-
Pacific Islander	27.00	27.00	-
Latino	7.00	11.00	-
**Parity**	1.13(1.47)	1.15(1.32)	NS
**Gravida**	2.67(1.94)	2.54(1.58)	NS
**Maternal Education Level**			NS
High School	66	92	-
College	23	34	-
Master	1	1	-
Unknown	5	2	-
**Ln Telomere Length**	5.53 (0.43)	5.41(0.38)	0.018 *
**IUGR(n)**	19.00	0.00	4.04e-7***
**Macrosomia(n)**	1.00	11.00	0.031*
**Chronic Hypertension (n)**	24.00	11.00	0.0013**
**History of Asthma (n)**	16.00	19.00	NS
**History of Anemia (n)**	26.00	15.00	0.0046**
**Baby sex (n)**			NS
Male	54.00	61.00	-
Female	41.00	68.00	-
**Membrane ruptured(n)**	21.00	51.00	0.0089**

*Categorical variables are compared with Chi-square test. Numeric variables are compared with t-test. Note: the test is based on each single variable, with no confounding adjustment.

*Significance level: * <0.05, ** <0.01, ***<0.001.

### Placental TL is not associated with severe PE but with gestational age

Although placental TL is longer in the severe PE group from the exploratory analysis (Table 1), such difference may be due to other confounding factors, such as delivery gestational age. To explore potential confounders of PE, we examined the effect of all other clinical variables on PE using logistic regression and reported the p-values of top variables in Figure 1B and Supplementary Table 1. Indeed, the P-values show that delivery gestational age has the strongest relationship with severe PE (p=4.25e-08, Figure 1B). Gestational age-adjusted TLs in each race, blood type, maternal age interval, and infant sex subgroup show no significant difference between severe PE and controls (Supplementary Figure 3). Moreover, when examining the contribution of all 25 phenotypic variables to TL, other demographic variables present no significant difference among racial variation, delivery gestational age shows the highest and most dominant proportion variance of 4.18% explained according to the ANOVA test, much higher than that of 2.09% from the 2nd ranked Latin ethnicity. Severe PE is not ranked among the top 10 variables (Figure 1C). Thus, the exploratory analyses coherently show the important relationship between gestational age and TL change in the third trimester.

To quantitatively delineate the relationship between TL and phenotypic factors including severe PE, we then constructed a full linear regression model by regressing placental TL on all 25 demographic and clinical variables and significant pairwise interactions among them, using both severe PE and control samples (n=224). This full model shows no significant difference (P = 0.462) in TL between severe PE patients and controls (Supplementary Table 2). We next determined the optimal model (Table 2), by applying the stepwise selection algorithm with the lowest Akaike Information Criterion (AIC) on the full model. Again, severe PE was excluded from the optimal model due to its lack of contribution to model fitness. Instead, placental TL shows a negative association with gestational age (beta = -0.03, p = 9.94e-4) according to the optimal model (Figure 1D and Table 2). The consistent decrease in TL is correlated with the active cell divisions in the placenta through the last trimester of pregnancy.

**Table 2:** The optimal linear regression model results using 224 samples.

**Variable name**	**Beta**	**Standard error**	**P-value**	**Significance**
**(Intercept)**	6.607	0.360	< 2e-16	***
**Mothers’ Age**	0.001	0.004	0.83577	
**Gestational Age at Delivery (Week)**	-0.030	0.009	0.00099	***
**Vaginal Bleeding**	0.118	0.068	0.08611	.
**Blood Type O**	-0.119	0.053	0.02498	*
**Latin**	0.281	0.096	0.00397	**
**Pacific Island**	0.095	0.062	0.12897	
**Parity**	-0.039	0.020	0.05237	.

*Significance level: * <0.05, ** <0.01, ***<0.001.

To better control the confounding effect of delivery gestational age on TL, we focused on the subset of severe PE patients (n=18) with comparable delivery gestational age (gestational age greater or equal to 38 weeks) to the control samples (n=128). Boxplot shows no significant difference (P =0.73) in TL between full-term severe PE and controls (Figure 1E). The linear regression model of TL on all 26 phenotypic variables on this subset (Supplementary Table 3) again shows a lack of association between severe PE and TL (P =0.65). Additionally, we performed a gestational week stratified (weeks 38, 39 and 40+) comparison between full-term severe PE samples (n = 18) and their 1-on-1 matched controls (n = 18) based on delivery gestational age, ethnicity, maternal age and BMI. Again, boxplots show no significant difference (P = 0.35, P= 0.28, and P= 0.88) of TL between cases and controls in all gestational week strata (Figure 1F). The optimal model using only severe PE cases still shows that delivery gestational age is negatively (beta = -0.029, p= 0.047) associated with TL (Table 3 and Figure 1D). In all, our analyses from many different perspectives all show that the shorter telomeres observed in severe PE patients are mostly associated with the gestational ages rather than severe PE itself.

**Table 3:** The optimal linear regression model results using 95 severe PE cases.

**Variable name**	**Beta**	**Standard error**	**P-value**	**Significance**
**(Intercept)**	5.751	0.700	1.89E-12	***
**Mothers’ Age**	0.004	0.006	0.55905	
**Gestational Age at Delivery (week)**	-0.030	0.014	0.04222	*
**Vaginal Bleeding**	0.262	0.151	0.08631	.
**Mothers Height**	0.138	0.093	0.1421	
**Chronic Hypertension**	-0.146	0.099	0.14163	
**Blood type A**	0.168	0.094	0.07752	.
**Caucasian**	-0.319	0.120	0.00935	**
**Latin**	0.264	0.164	0.11228	

*Significance level: * <0.05, ** <0.01, ***<0.001.

### Racial disparity is present in placental TL

**Figure 2:**
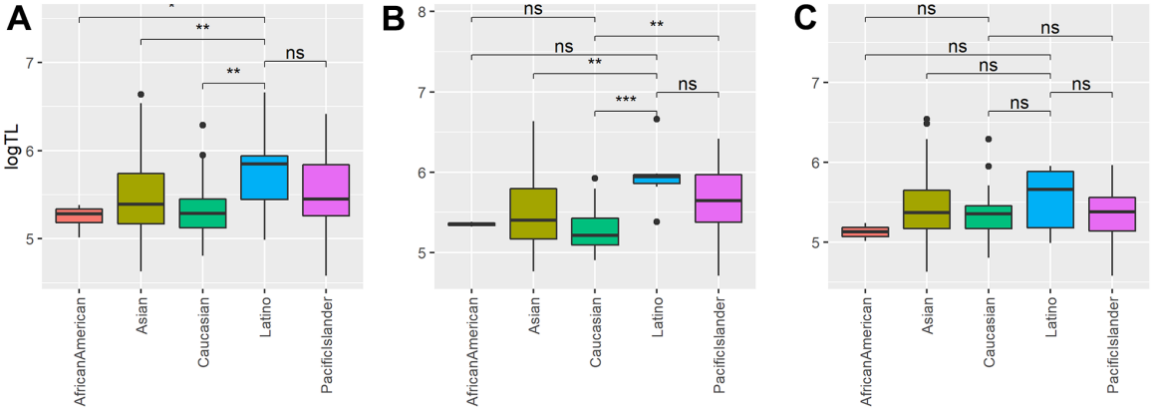
**The associations between placental telomere length (TL) and race.** Shown are the boxplot of placental TLs in Latino, Caucasian, African American, Pacific Islander and Asians in: (**A**) all samples (n=224); (**B**) severe PE samples (n=95); (**C**) control samples (n=129). “**” denotes the t-test p-value is less than 0.01, “*” denotes the t-test p-value less than 0.05 and “NS” means the p-value greater or equal to 0.05.

Besides gestational age, the optimal model also revealed significant associations of placental TL to other demographic or clinical variables (Table 2). Among them, race shows significance second to the delivery gestational age. In particular, Latino mothers have longer placental TL than non-Latino mothers (beta = 0.281, P = 3.97e-3) in combined severe PE and control samples, after adjusting for all other confounders with linear regression (Figure 2A). To further decouple the effect of severe PE on race-associated TL differences, we separated the severe PE cases and controls and built optimal models within each category, respectively.

Interestingly, the trend of longer placenta TL among Latino mothers still exists among severe PE patients (Table 3 and Figure 2B), though slightly above the statistical significance threshold (beta =0.264, P= 0.112). On the other hand, among control samples, Latino is no longer associated with TL (Table 4 and Figure 2C). This indicates that severe PE samples are the main contributors to the observed positive association of Latino ethnicity to TL overall. We also observe some other apparent racial associations in severe PE and control samples, respectively. Among severe PE patients, Caucasian mothers have shorter TL in the optimal model (beta = -0.319, P= 0.009), and a similar shorter TL trend exists with African American mothers among the healthy controls (Table 4 and Figure 2C), although not statistically significant (beta = -0.444, P= 0.093). We also compared maternal age, gestational age, maternal education level, parity and gravida among different ethnic groups (Supplementary Figure 4). No significant difference was found, suggesting low confounding effects from these variables for observed ethnicity association to TL. While PE itself does not appear to be associated with telomere length in each ethnicity statistically, the trends of difference of TL between PE vs controls are different among the ethnicities after adjusting for gestational age, the most major determinant of TL (Supplementary Figure 3). This suggests that there is interaction between ethnicity and PE. Overall, the data show that there exist racial differences in placenta TL, and such differences may be dependent on the severe PE status.

**Table 4:** The optimal linear regression model results using 129 control samples.

	**Beta**	**Standard error**	**P-value**	**Significance**
**(Intercept)**	5.624	0.151	<2e-16	***
**Mothers’ Age**	-0.004	0.005	0.4147	
**Chronic Hypertension**	0.221	0.118	0.063	.
**History of Asthma**	-0.229	0.090	0.0127	*
**African American**	-0.444	0.262	0.0931	.
**Macrosomia**	-0.189	0.114	0.1014	
**Parity**	-0.045	0.026	0.0881	.

*Significance level: * <0.05, ** <0.01, ***<0.001.

## DISCUSSION

The shorter TL is an indicator of stress and aging. Placenta TL is a molecular factor contributing to the rupture of the amniotic sac, onset of labor, and parturition [23]. Shorter placental TLs have been linked to placenta aging, oxidative stress and adverse pregnancy outcomes [24]. The study here addressed the possible (or lack of) associations between placental TL with severe PE and other physiological and clinical phenotypes, using the largest multiracial cohort so far. In particular, the subjects include relatively large Asian (n = 117) and Pacific Islander populations (n=54), who were less studied in previous research compared to other races. The most major finding of this study is that placental TL shortening is not significantly associated with severe PE; rather, it shortens significantly as gestational age increases.

PE and shorter gestational age are like horse and carriage, which go hand in hand. Any virginal preterm deliveries are not “healthy controls”, by definition. One has to get around the impossible scenario of “preterm healthy controls” by computational approaches: either using linear regression to “project” what might happen in preterm or stratifying the samples by gestational ages among the “term deliveries”. By applying these analytical techniques, our study puts the long-time debate whether severe PE affects placental TL to the rest. The data analysis shows the lack of association between severe PE and placental TL, even though the severe PE samples do have longer TL. The apparent longer placenta TL in severe PE is mostly attributed to earlier gestational ages that accompany the preterm deliveries of babies from severe PE patients [25], rather than severe PE itself. That is, when the placenta grows with the gestational age, cells divide and telomeres shorten [26].

Support to the conclusion above comes from the quantitative multiple regression model, where the other potential confounders are considered. The lack of association between severe PE and placental TL agrees with several previous findings, where TL was measured by qPCR and telomere restriction fragments (TRF) method [16, 17] and qPCR [18]. The study showing a negative association between severe PE and placental TL had a smaller sample size (14 severe PE cases, 20 controls) [15] and didn’t report or adjust for other confounders, though gestational age (36 +/- 1.41 for the severe PE, 37.15+/-3.9 for the controls) is less of a concern. It also utilized a different method Quantitative Fluorescence *in situ* hybridization (Q-FISH) for TL measurement. qPCR and TRF are the most common telomere measurement methods in research and commercial settings. Q-FISH provides high resolution but is labor intensive and thus is not convenient for large-scale datasets [27]. These differences in measurement approaches and statistical rigor may contribute to their different conclusions.

This study also shows the associations of TL with race. In particular, we observed that the placentas of Latino mothers have longer placental TL compared to non-Latino mothers, after adjusting for other possible confounders by linear regression. This association still exists in severe PE samples, but not within healthy controls. Previous studies found longer TLs in blood samples of Hispanic adults compared to white adults but no significant difference in infants [28, 29]. To our knowledge, the longer TL in placentas associated with Latino mothers, particularly those with severe PE, is reported here for the first time. Additionally, we also found that Caucasian mothers have significantly shorter placenta TL in severe PE samples, whereas in healthy control populations the placenta TL from African American mothers tend to be shorter. Corroborating our latter observation, a previous study also found shorter placental TL in black mothers compared to white mothers [23]. While future work is needed to explore more mechanisms of racial disparity of TL, our result shows evidence of racial disparity manifested in placental TL, which may be a source of birth outcome disparities. Race has been linked to the incidence rate, severity, and onset time of PE. Most previous research on the association between PE and TL contains only white participants [17] or didn’t adjust for racial difference [15, 16, 18]. This study shows that clearly, it is important to use multi-racial cohorts and statistical adjustment to ping-point racial differences.

Several caveats should be noticed in this study. Despite the strong evidence of the lack of association between severe PE and TL, our study measures the TL using bulk placenta tissue samples. The compositions of the cell types and their TL differences are also other possible sources of variations. As the placenta biology is stepping into single cell era, technologies such as single cell sequencing that identifies cell types and proportions, and ΩqPCR method that determines absolute TL from single-cell level [30], could further reveal the heterogeneity of TL that are masked by the current tissue level measurements. Another limitation is that we could not stratify the PE patients studied here by its onset time given the lack of such information in the original database. As a heterogeneous disease, early-onset PE (occurs before gestational week 34) has different pathophysiology from late-onset PE. Similarly, information such as income and physical activity data were not recorded originally to allow further investigation for confounding. Though we could not obtain sample storage time, it should not be a concern for observed TL length variation due to DNA quality inconsistence. Previous literature showed biobank samples in frozen tissue at cryogenic temperatures had no time-dependent decrease in tissue RNA or DNA quality, after long-term storage (>11 years) [31]. Lastly, the study here focused on severe PE, rather than mild PE, thus the effect of mild PE on TL cannot be investigated. However, given the more severe phenotype, it may be reasonable to speculate that mild PE is not associated with TL either.

In summary, our study shows no significant telomere change in severe PE-complicated placentas compared to healthy controls. Rather, TL shortening is mostly associated with gestational ages. Additionally, placenta TL also demonstrates race related disparities, in both severe PE and healthy samples.

## MATERIALS AND METHODS

### Biospecimens

Placenta samples from 224 women (95 with severe PE and 129 full-term healthy controls), who delivered their babies in Hawaii between January 2006 and June 2013 were collected and analyzed in this study. Samples were delivered at Kapiolani Women and Children’s Hospital from and stored at the University of Hawaii Biorepository (HiBR). HiBR is one of the largest research tissue repositories in the pacific region, containing specimens from more than 9250 mother-child pairs at the time of sample collection. Severe PE was defined as sustained pregnancy-induced hypertension with blood pressure higher than 160/110 mmHg and urine protein and/or organ dysfunction according to the diagnosis of OBGYNs in Kapiolani Medical Center. For each severe PE case, a matched healthy control sample was identified using mother age, prepregnant body mass index (BMI) and ethnicity group as the matching criteria. All other clinical information was recorded and analyzed for potential confounding factors. Samples from smoking are removed due to the small size. Samples with gestational diabetes and neonatal malformations (which only occurred in the case group) were excluded from the data set, to avoid potential sources of confounding.

### TL measurement

Placenta samples were obtained from the intermediate portion of the placenta after maternal and fetal membranes were trimmed away. The placenta tissue samples were stored at -80° C. The genomic DNA extracted from placenta tissue was performed using AllPrep DNA/RNA/Protein Mini Kit (Qiagen, USA) according to the manufacturer’s instructions. Briefly, 30mg of frozen placenta tissue samples were weighted in a 50 ml Falcon tube (VWR, USA) and mixed with 600 μl buffer RLT (Qiagen, USA) supplemented with 1% β-mercaptoethanol (Sigma, USA). The tissue was then homogenized with Tissue Ruptor II (Qiagen, USA) for 30 seconds at medium speed. The lysate was transferred to a 2 ml microcentrifuge tube and centrifuged for 3 minutes at 13,000g at 4° C. Then the supernatant was pipetted into an AllPrep DNA spin column (Qiagen, USA) and processed following Kit’s instruction. The DNA concentration and purity were evaluated by NanoDrop2000 spectrophotometer (Thermo Scientific, Waltham, USA) and the DNA integrity was investigated by electrophoresis in 1% agarose gel with ethidium bromide (Sigma, USA).

The average TL of placenta tissue samples was measured using the Absolute Human TL Quantification qPCR assay Kit (AHTLQ, ScienCell Research Laboratories, USA). Each sample was tested in duplicate with StepOne™ Real Time System (Applied Biosystems, USA) using 96-well reaction plates. The melting curve was observed to evaluate the specificity and efficiency of the qPCR reaction. The Comparative ∆∆Cq (Quantification Cycle Value) method was applied to calculate the relative average TL of target tissue samples [32]. The coefficient of variation (CV), the ratio of standard deviation over mean, is used as the metric to measure performance precision among all technically duplicated samples in qPCR. The maximum CV among samples is 0.105, smaller than the recommended cutoff threshold 0.11. A 100 bp region single copy reference (SCR) primer set on chromosome 17 was used as the reference. The absolute TL is calculated as the relative TL multiplied by the SCR TL.

### Demographical, clinical and physiological data

A total of 26 demographical, clinical and physiological variables obtained from the University of Hawaii HiBR biobank were used in this study: mother’s age, height, pre-pregnancy BMI, parity, gravida, maternal education level high school, college, masters and unknown with college as the reference level, weight gain during pregnancy, blood type A, B, O, AB with AB as the reference level, African American race, Asian race, Caucasian race, Latino race and Pacific Islander with Asian race as the reference level, history of asthma, history of anemia, chronic hypertension, macrosomia, vaginal bleeding, IUGR, membrane ruptured, babies’ sex, gestational age at delivery, and sample group. Categories with no more than 5 patients (eg. amnion infection) were too low in frequency to draw any sounding conclusion, thus we removed them from our analysis. The missing values were imputed with the predictive mean match (PMM) algorithm from R package “mice” [33].

### Statistical analysis

Two-sided Welch t-tests and Chi-squared tests were used to compare numeric and categorical characteristics in case and control groups. Logistic regression was performed to measure the correlations between severe PE and other factors. The proportional variance explained (PVE) values of all variables were calculated using eta-values from the 1-way ANOVA result. The PVE value describes the proportion of total variance in data that can be explained by a certain variable, demonstrating its importance [34]. The absolute TL was ln-transformed to reduce skewness and ensure normality. TL refers to ln-transformed placental TL unless stated otherwise.

A multiple linear regression model was constructed by regressing TL of 224 samples on all 26 variables and the most significant interaction between gestational age and infant sex. We refer to this model as the full model. To select the most relevant variables and improve model fitness, we applied the stepwise selection algorithm on the full model to search for the optimal model with the lowest Akaike Information Criterion (AIC), by adding or removing one variable at a time. This stepwise selected model is referred to as the optimal model, including 7 variables: mother’s age, gestational age at delivery, baby’s sex, blood type O, Latino ethnicity, Pacific Islander ethnicity and parity. To assess the impact of severe PE without bias from different gestational ages, we stratified the TL of severe PE patients exceeding 38 weeks of gestation (n = 18) and a one-on-one matched control subset (n=18) by gestational week at birth. Each sample from the control subset was matched to one full-term severe PE case based on the maternal age, race, gestational age and BMI. We then compared the TL in each gestational week group with a t-test.

Two multiple linear regression models were constructed on severe PE cases and controls respectively by regressing TL on all other variables. To select the most relevant variables and improve model fitness, we applied the stepwise selection algorithm on these two models again to search for the optimal models with the lowest Akaike Information Criterion (AIC), by adding or removing one variable at a time until AIC cannot be further reduced.

All analyses were conducted with different packages in R software version 4.0.2 [35]. “tidyr”, and “dplyr” were used in data cleaning [36, 37]; “mice” was used to impute missing values [33]; “ggplot2”, “gridExtra”, “ggsignif” and “corrplot” were used in visualization [38–41]. All R script used in this project is in repository.

## Supplementary Material

Supplementary Figures

Supplementary Tables
