# Supplementary material for: Placental telomere length shortening is not associated with severe preeclampsia but the gestational age
Source: Aging (Albany NY). 2022 Dec 27;15(2):353–70. doi: 10.18632/aging.204445 (PMC9925682; doi:10.18632/aging.204445)
Supplement: Supplementary Figures [file aging-15-204445-s001.pdf]

SUPPLEMENTARY FIGURES

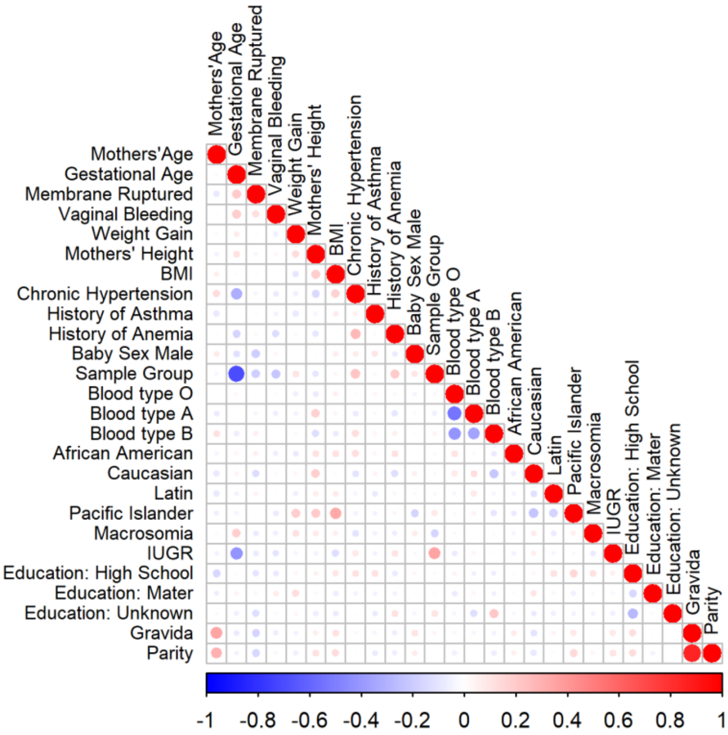

Supplementary Figure 1. The Pearson's correlation heatmap of all 26 variables. PE(sample group) and gestational age show strong negative correlation.

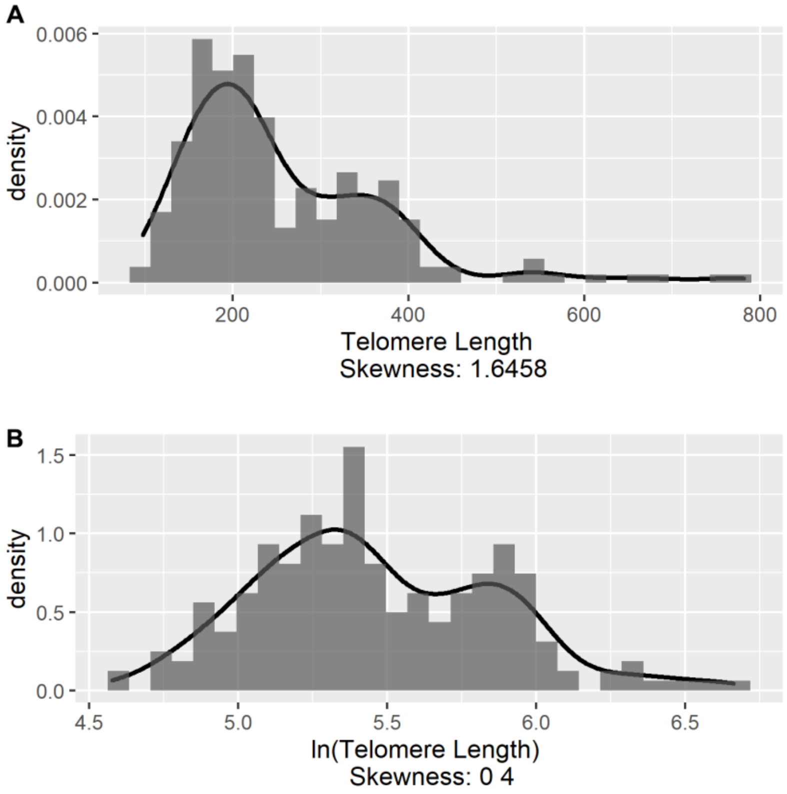

Supplementary Figure 2. The histograms and density plots of placental TL before and after ln-transformation. (**A**) The TL density distribution before ln-transformation. Distribution is skewed. (**B**) The TL density distribution after ln-transformation. The distribution is more symmetric.

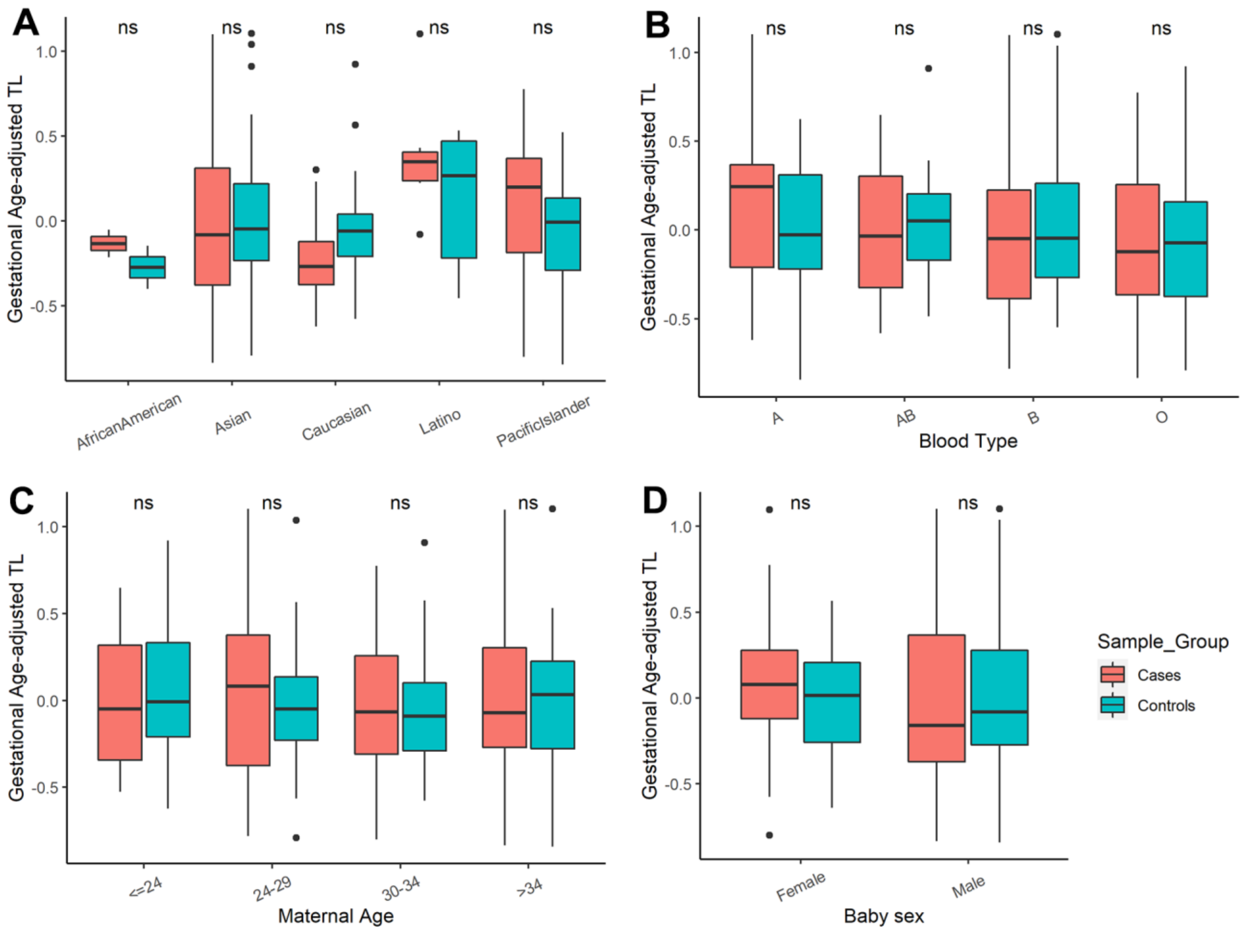

Supplementary Figure 3. Gestational age-adjusted TLs in each subgroup show no significant difference between PE and controls. (**A**) Gestational age adjusted TL by sample group in each racial group. (**B**) Gestational age adjusted TL by sample group in each blood type subgroup. (**C**) Gestational age adjusted TL by sample group in each maternal age group. (**D**) Gestational age adjusted TL by sample group in different baby sex group

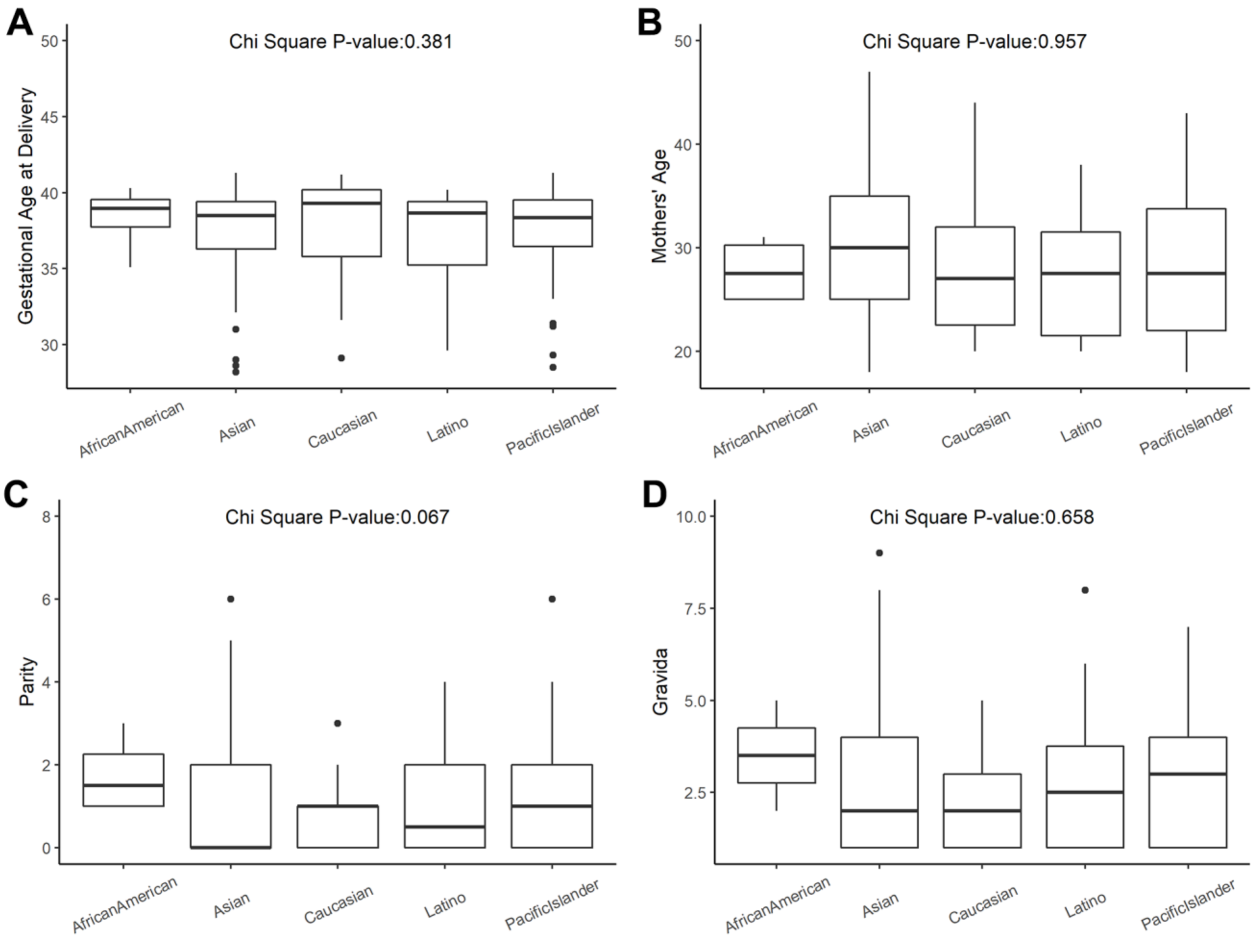

Supplementary Figure 4. No significant difference in (**A**) maternal age, (**B**) gestational age at delivery, (**C**) gravida and (**D**) parity is found among racial groups.
