# Supplementary material for: Placental telomere length shortening is not associated with severe preeclampsia but the gestational age
Source: Aging (Albany NY). 2022 Dec 27;15(2):353–70. doi: 10.18632/aging.204445 (PMC9925682; doi:10.18632/aging.204445)
Supplement: Supplementary Tables [file aging-15-204445-s002.pdf]

SUPPLEMENTARY TABLES

Supplementary Table 1. Logistic regression of all other clinical variables on PE.

Variable name	Coefficient	Standard error	P-value	Significance
**(Intercept)**	71.78	13.74	0.00	***
**Mothers' Age**	0.03	0.05	0.51	
**Gestational Age at Delivery (Week)**	-1.85	0.34	0.00	***
**Membrane Ruptured**	-0.28	0.59	0.63	
**Vaginal Bleeding**	-0.93	0.74	0.21	
**Weight Gain(lbs)**	0.05	0.02	0.00	**
**Mothers' Height**	-0.62	0.73	0.40	
**Pre-pregnancy BMI**	0.05	0.05	0.30	
**Chronic Hypertension**	-0.06	0.99	0.95	
**History of Asthma**	0.20	0.76	0.79	
**History of Anemia**	0.65	0.79	0.41	
**Baby Sex**	0.21	0.59	0.72	
**Blood Type O**	-2.11	1.14	0.06	.
**Blood Type A**	-2.31	1.14	0.04	*
**Blood Type B**	-2.77	1.28	0.03	*
**African American**	3.24	1.67	0.05	.
**Caucasian**	1.34	1.03	0.19	
**Latin**	0.07	1.14	0.95	
**Pacific Island**	0.43	0.79	0.59	
**Macrosomia**	0.40	1.23	0.74	
**IUGR**	16.90	1619.38	0.99	
**Education: High School**	-0.22	0.71	0.76	
**Education: Masters**	-0.29	1.69	0.86	
**Education: Unknown**	0.52	2.43	0.83	
**Gravida**	0.03	0.36	0.93	
**Parity**	-0.58	0.49	0.24	

Supplementary Table 2. Linear regression results using 27 variables and all 224 samples.

Variable name	Coefficient	Standard error	p-value	Significance
**(Intercept)**	6.371	0.654	<2e-16	***
**Mothers' Age**	-0.001	0.005	0.880	
**Gestational Age at Delivery (Week)**	-0.021	0.015	0.149	
**Membrane Ruptured**	-0.035	0.062	0.572	
**Vaginal Bleeding**	0.116	0.073	0.115	
**Weight Gain(lbs)**	0.000	0.002	0.922	
**Mothers' Height**	0.007	0.072	0.920	
**Pre-pregnancy BMI**	-0.001	0.005	0.784	
**Chronic Hypertension**	0.006	0.087	0.949	
**History of Asthma**	-0.105	0.076	0.172	
**History of Anemia**	-0.016	0.075	0.834	
**Baby Sex**	-0.014	0.057	0.804	
**PE Cases**	0.060	0.081	0.462	
**Blood Type O**	-0.105	0.109	0.333	
**Blood Type A**	0.022	0.110	0.844	
**Blood Type B**	-0.012	0.117	0.915	
**African American**	-0.108	0.215	0.617	
**Caucasian**	-0.104	0.090	0.251	
**Latin**	0.230	0.109	0.036	*
**Pacific Island**	0.058	0.082	0.482	
**Macrosomia**	-0.102	0.124	0.413	
**IUGR**	0.002	0.110	0.989	
**Education: High School**	0.007	0.068	0.914	
**Education: Masters**	-0.124	0.295	0.675	
**Education: Unknown**	-0.190	0.168	0.259	
**Gravida**	0.016	0.031	0.608	
**Parity**	-0.055	0.038	0.148	

Supplementary Table 3. Linear regression result on full-term (gestational age > 38 week) samples (n=147).

Variable name	Beta	Standard error	P-value	Significance
(Intercept)	7.91	1.66	0.00	***
Mothers' Age	0.00	0.01	0.76	
Gestational Age at Delivery (Week)	-0.05	0.04	0.22	
Membrane Ruptured	-0.03	0.07	0.66	
Vaginal Bleeding	0.10	0.08	0.23	
Weight Gain(lbs)	0.00	0.00	0.74	
Mothers' Height	-0.06	0.10	0.56	
Pre-pregnancy BMI	0.00	0.01	0.48	
Chronic Hypertension	0.14	0.13	0.28	
History of Asthma	-0.21	0.10	0.03	*
History of Anemia	0.11	0.10	0.29	
Baby Sex	0.05	0.07	0.48	
PE Cases	0.05	0.11	0.65	
Blood Type O	-0.19	0.14	0.16	
Blood Type A	-0.07	0.14	0.60	
Blood Type B	-0.13	0.15	0.39	
African American	-0.01	0.25	0.96	
Caucasian	0.04	0.11	0.74	
Latin	0.11	0.13	0.40	
Pacific Island	0.02	0.10	0.85	
Macrosomia	-0.11	0.12	0.37	
IUGR	0.08	0.43	0.85	
Education: High School	0.07	0.08	0.38	
Education: Masters	-0.15	0.29	0.60	
Education: Unknown	-0.19	0.30	0.54	
Gravida	-0.01	0.05	0.91	
Parity	-0.05	0.06	0.45	
